# Color ensembles: Sampling and averaging spatial hue distributions

**DOI:** 10.1167/jov.20.5.1

**Published:** 2020-05-11

**Authors:** Lari S. Virtanen, Maria Olkkonen, Toni P. Saarela

**Affiliations:** 1 Department of Psychology and Logopedics, Faculty of Medicine, University of Helsinki, Helsinki, Finland; 2 Department of Psychology, Durham University, Durham, UK

**Keywords:** color, spatial integration, ensemble perception, modeling

## Abstract

Color serves both to segment a scene into objects and background and to identify objects. Although objects and surfaces usually contain multiple colors, humans can readily extract a representative color description, for instance, that tomatoes are red and bananas yellow. The study of color discrimination and identification has a long history, yet we know little about the formation of summary representations of multicolored stimuli. Here, we characterize the human ability to integrate hue information over space for simple color stimuli varying in the amount of information, stimulus size, and spatial configuration of stimulus elements. We show that humans are efficient at integrating hue information over space beyond what has been shown before for color stimuli. Integration depends only on the amount of information in the display and not on spatial factors such as element size or spatial configuration in the range measured. Finally, we find that observers spontaneously prefer a simple averaging strategy even with skewed color distributions. These results shed light on how human observers form summary representations of color and make a link between the perception of polychromatic surfaces and the broader literature of ensemble perception.

## Introduction

**Figure 1.**
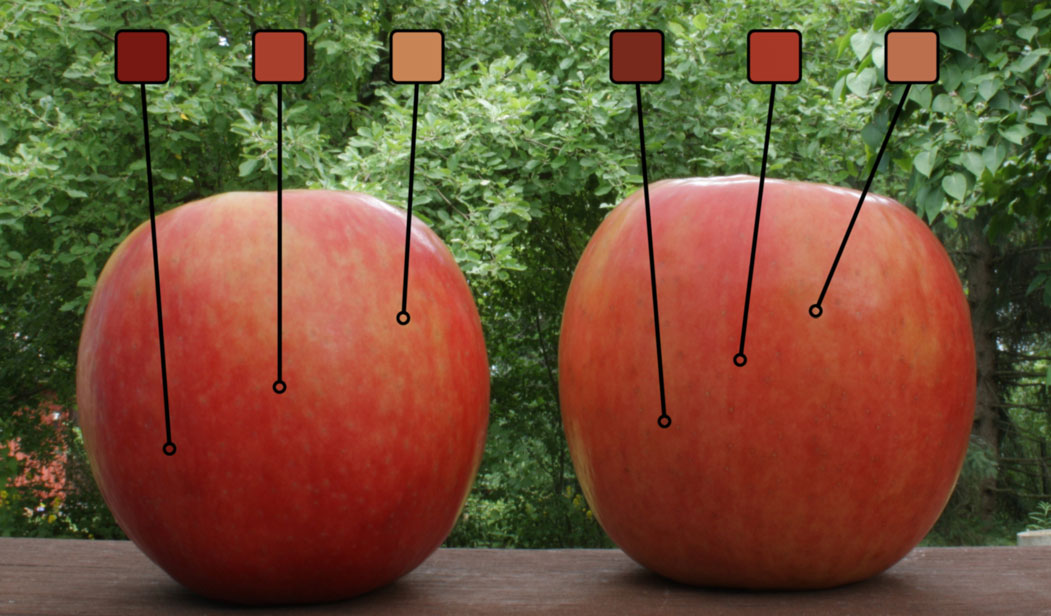
A photograph of two apples with insets showing colors in different locations on the surface of the apples. Despite the large range of surface colors, most observers have no trouble assigning a representative color to these apples.

How do we attribute the color red to an apple? Objects often appear to have one predominant color, although their surfaces may contain substantial spatial variation in how they reflect wavelengths of light. Consider the apples in Figure 1 and the range of different colors shown in the insets. Parts of the apple reflect more long-wavelength light, appearing mostly reddish, while some parts reflect light more evenly across the wavelength spectrum, appearing more yellowish or brownish. Despite this variety in the wavelengths of reflected light, we probably classify both apples as red and may determine that the one on the left is the redder of the two.

To form an estimate of the “representative” color—perhaps the mean—of one of the apples in Figure 1, the visual system has to integrate color information from different spatial locations. Summarizing information across several samples is generally useful as it reduces noise in perceptual estimates (Alvarez, 2011), making it a potentially beneficial strategy across sensory domains. For a wide variety of visual stimulus attributes, distinct items can be swiftly and effortlessly integrated from several parts of the visual field into something akin to a statistical representation (for reviews, see Alvarez, 2011; Whitney & Leib, 2018). The visual system is able to form ensemble percepts for a wide variety of visual stimulus attributes ranging from orientation (Parkes et al., 2001) to facial emotions (Haberman & Whitney, 2007, 2009).

Although spatial variation in surface color is the norm, we know relatively little about integration of color information across space. A small number of studies have investigated the extraction of summary descriptions from natural polychromatic surfaces (Milojevic et al., 2018; Vurro et al., 2013), multicolor mosaics (Kimura, 2018; Kuriki, 2004), and hue ensembles (Maule & Franklin, 2015, 2016; Maule et al., 2014; Webster et al., 2014). Milojevic et al. (2018) showed that human observers use the mean hue (or possibly the colorimetric mean) when sorting autumn leaves by color. This is also true for more simplistic color arrays (Maule et al., 2014; Webster et al., 2014), although there is a tendency to deviate toward the most saturated sample in a color mosaic (Kimura, 2018; Kuriki, 2004).

But how efficiently do observers use information in a multicolored array? Maule and Franklin (2016) estimated that observers only use as few as two samples in a 16-element hue array when judging average color. This is a remarkably low estimate, compared to those from other visual feature dimensions such as orientation and motion where the number of samples utilized by the visual system grows as a function—in some cases roughly as the square root—of the number of samples available (Dakin, 2001; Dakin et al., 2005; also see Figure 4 in Whitney & Leib, 2018). No studies focusing on color, however, have systematically manipulated the number of elements (and noise) in the stimulus, which might be necessary to adequately quantify the efficiency of spatial integration.

Finally, previous research on color ensembles has mostly focused on the perceived average color of a symmetric color distribution. There is evidence that the shape of a color distribution can be implicitly represented in certain tasks (Chetverikov et al., 2017). As of yet, we do not know how the shape of a color distribution affects the perceived color of an ensemble stimulus, if at all.

Here we systematically investigate the rapid extraction of summary information from color ensembles. We use an approach similar to one that has been used to study the integration of orientation signals in the human visual system (Dakin, 2001). In natural viewing, object color varies along multiple dimensions (lightness, saturation, and hue). We focus here on the hue dimension of color. Hue variation is highly prevalent in most natural surfaces, while it may be less variable in man-made objects. It is arguably the most informative aspect of color in terms of underlying surface pigment (Kingdom, 2008), categorization by color (Milojevic et al., 2018), and object identity (see Figure 3 in Olkkonen et al., 2008). In order to estimate the efficiency in extracting hue information from ensembles, we systematically vary the external noise (variance of the hue distribution from which the stimuli are drawn) and the number of samples available to the observer and estimate effective sampling through mathematical modeling. We find that observers use a vastly larger number of samples than previously estimated for color. In a control experiment, we dissociate the effects of number of samples and total stimulus area in the visual field. Surprisingly, hue averaging performance is equivalent whether the stimulus elements are abutting or spatially separated, suggesting a greater role for surface as opposed to edge information. Finally, we show that the spontaneous integration strategy for most observers is to use a simple average when the shape of the hue distribution is varied.

## General methods

We conducted three experiments to characterize different aspects of the spatial integration of hue. Below, we present the general methods that apply to all three experiments unless otherwise noted. Methods specific to each experiment are presented in their respective sections.

### Observers

Twelve observers took part in the experiments (eight female, age range 20–60, mean age 33 years). Not all observers participated in all experiments; Table 1 lists the participants for each experiment. Two observers were the authors LSV and TPS; the other 10 observers were naïve to the theoretical aims of the experiment. All observers had normal or corrected-to-normal visual acuity and normal color vision as assessed by the Ishihara color plates (Ishihara, 1987). Observers gave informed consent and received cinema tickets for their time. The experimental protocol was approved by the University of Helsinki Ethical Review Board in Humanities and Social and Behavioral Sciences.

**Table 1.** List of observer participation in different experiments. Observer 12 was omitted from analysis because psychometric functions could not be fit to the data for several experimental conditions.

	O1	O2	O3	O4	O5	O6	O7	O8	O9	O10	O11	O12	Total
Experiment 1	x	x	x				x	x	x	x	x	(x)	8 (9)
Experiment 2	x			x	x		x	x	x	x	x		8
Experiment 3	x	x	x	x		x	x			x	x		8

One of the nine observers who participated in Experiment 1 reported not being able to determine hue differences toward “bluer” and “yellower” in the stimuli, which was essential to the task. This difficulty persisted even after the observer was shown a continuum from yellow to blue. For some of the experimental conditions, a psychometric function could not be fit, so this observer was omitted from further analysis. After this omission, each experiment had eight participants.

### Apparatus

The experiments were conducted on an HP Z230 Desktop PC running MATLAB (Version R2016b, Build 9.1.0.441655) with the PsychToolBox-3 extensions (Brainard, 1997; Kleiner et al., 2007; Pelli, 1997). Stimuli were presented on a 23 inch. ViewPixx monitor controlled by an Nvidia Quadro K620 graphics card. The monitor resolution was 1,920 × 1,080 pixels, with 100-Hz refresh rate, 10-bit color channels, and a maximum luminance of 250 cd/m^2^. The display white point was set to be metameric to D65. The display primary spectra and gamma functions were measured using the X-Rite i1Pro spectrophotometer, and linearization of luminance was achieved by interpolating the gamma functions. Observers took the experiment in a dimmed room, and their viewing distance was held constant at 90 cm from the screen using a chinrest. Observers gave their responses using a regular keyboard.

### Stimuli

We defined stimulus chromaticities in the CIELAB color space with the monitor white point as the reference. Stimulus lightness and chroma were held constant at L = 60 and C = 50, while hue varied on the hue circle in the CIELAB space. To ensure good visibility of the color stimuli, the background was set to a slightly darker uniform gray (luminance 46 cd/m^2^, L = 50). As hue averaging is compromised over excessively large variations (Maule & Franklin, 2015), we chose to limit hue variation within “greenish” colors. To enable a choice task between “bluer” and “yellower” categories, we employed hues around the center point of 150 degrees of hue angle, which is roughly in the middle of the green category (Bae et al., 2015).

The standard stimulus consisted of a square grid with a varying number of square elements (from 1 to 64) near the middle of the screen. The mean color of the standard stimulus was randomized on each trial between 140 and 160 degrees of hue angle, so that the observer would have to estimate the average hue of both the standard and the comparison (see below) stimulus on every trial, instead of forming and relying on an “implicit standard” based on the standard stimulus mean. The stimulus center location was randomized around the screen center within a 60-pixel range from the screen midpoint to avoid local adaptation to edges and color aftereffects. Each element was filled with a uniform color and extended one degree of visual angle. Depending on the experimental condition, the elements were either abutting or separated by a $\frac{1}{3}$-degree gap of background gray. For no-noise trials, all stimulus elements shared the standard stimulus mean hue, making the whole stimulus uniform in hue. For noise trials, a set of random hue angles for the elements was drawn from a circular von Mises distribution centered at the standard stimulus mean hue. The von Mises distribution's probability density function for angle x is of the form:

(1) $$\begin{array}{c} d\left(x|\mu ,\kappa \right)=\frac{e^{\kappa \cos(x-\mu )}}{2\pi I_{0}\left(\kappa \right)},\; \end{array}$$

where $I_{0}\left(\kappa \right)$ is the modified Bessel function of order 0, μ is the mean, and κ is the concentration parameter. We used the values κ = 40 and κ = 15 for the low-noise and high-noise conditions, respectively (corresponding approximately to normal distributions with standard deviations of 9.12 and 14.79 degrees).

The comparison stimulus was spatially identical to the standard stimulus but had its mean hue drawn from a distribution centered at one of the nine comparison levels around the standard stimulus mean. The comparison levels were selected from preset ranges for each noise (e.g., Experiment 1) or set size (e.g., Experiment 2) condition. The decision was based on the practice results of individual observers so that the comparison stimulus covered the range that allowed measuring thresholds.

### Procedure

**Figure 2.**
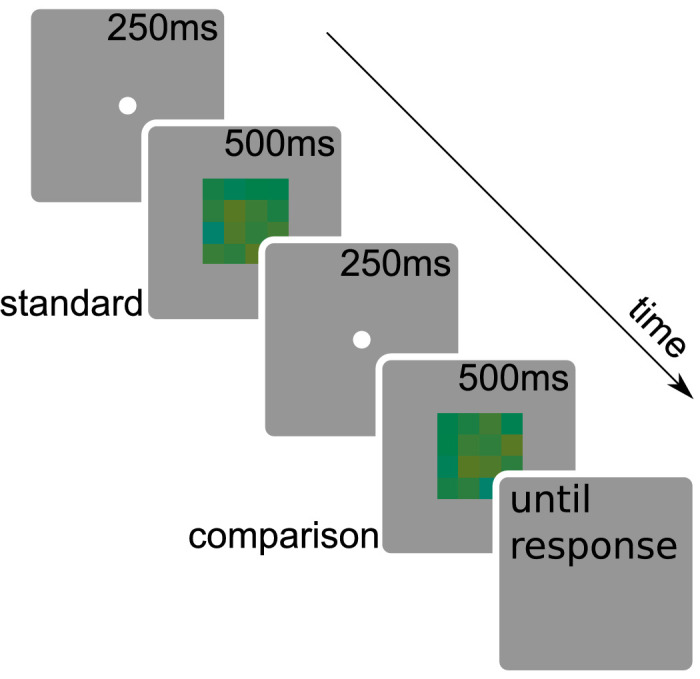
The time course of a single trial. The observer was shown a dot indicating the center of the screen for 250 ms, then the standard stimulus for 500 ms. This was followed by an inter-stimulus interval with the center dot for 250 ms and then the comparison stimulus for 500 ms. Finally, the screen remained blank until the observer gave a response indicating whether the second stimulus was yellower or bluer than the first. The following trial began after response.

The time course of one trial is illustrated in Figure 2. On each trial, the observer was shown a blank screen (uniform gray of the background) with a white dot of 0.1 degrees of visual angle indicating the middle of the screen for 250 ms. The observers knew that the white dot indicates the approximate location of the stimulus, but they were not required to maintain fixation exactly on the dot. Next, the standard stimulus was displayed for 500 ms. Again, the blank screen with a center dot was displayed for 250 ms, now followed by the comparison stimulus for 500 ms. Finally, the screen remained blank until the observer gave a response. The observer's task was to respond whether the hue represented by the whole of the comparison stimulus was “yellower” or “bluer” than that for the standard stimulus. We did not use the word “mean” in the instructions in order to avoid encouraging particular strategies (this was especially important for Experiment 3). The observer responded by pressing the left or right arrow key on the keyboard. The mapping between response and left/right key was counterbalanced across observers. Observers received no feedback for the correctness of their responses. Standard and comparison intervals were not randomized (always presented in a fixed order) for consistency across experiments, as randomization was not possible in some experiments.

### Data analysis

Data analysis was performed in MATLAB (Version R2018b, Build 9.5.0.944444), except for the Bayesian analyses of variance (ANOVAs) and *t*-tests, which were conducted in JASP (JASP Team, 2018). We recorded the number of “bluer” responses by the observer for each comparison level. A psychometric function (cumulative Gaussian) was fit to these response data by a maximum likelihood method with mean and standard deviation (*SD*) as parameters. The mean gives the point of subjective equality (PSE), and its difference from the true value gives an estimate of bias (used in Experiment 3). We used the *SD* as an estimate of the discrimination threshold (Experiments 1 and 2).

A bootstrap method was used to estimate the standard error of the mean (*SEM*) for individual observers in different experimental conditions (Efron & Tibshirani, 1986). The data were sampled with replacement for each data point, and a psychometric function was fit to the sampled data. This was repeated for 2,000 iterations, and the 68.27% confidence limits of the resampled parameters were drawn to represent ±1 *SEM*. Repeated-measures Bayesian ANOVAs and *t* tests were performed to test the effects of the particular experimental manipulations in each experiment.

### Modeling

In Experiment 1, external noise as symmetric and equal among stimulus elements. Internal noise was also assumed to be symmetric and equal, making simple averaging with equal weights an optimal strategy to estimate stimulus hue. To estimate the effective sampling (the number of stimulus elements the observer is able to use), we fit a noise model to each observer's data. The model is similar to one that has been used for modeling the integration of orientation signals (Dakin, 2001). The model observer averages hue samples with equal weights, matching the optimal strategy in the task. Internal noise in the model has a zero-mean Gaussian distribution, and it affects the coding of each sample equally and independently. The bottleneck for the model observer is limited capacity in sampling—not all samples can necessarily be used, making performance sub-optimal. The more samples the observers are able to utilize, the more they are able to average out noise in the stimulus. By introducing external noise and estimated internal noise to the equation, one can estimate how many individual cues are needed to average out enough noise to match the observer's performance. The basic form of the model is:

(2) $$\begin{array}{c} \sigma_{r}=\sqrt{\frac{{\sigma_{i}}^{2}+{\sigma_{e}}^{2}}{n_{s}}+{\sigma_{o}}^{2}},\; \end{array}$$

where $\sigma_{r}$ is the noise in the internal response to the whole stimulus, σ_e_^2^ is the external noise variance, σ_i_^2^ is the variance in the internal response to a single element, σ_o_^2^ are other sources of noise, and *n*_s_ is the number of samples used by the observer. In our experiment, values of $\sqrt{2}\sigma_{r}$ would indicate observer performance in terms of discrimination thresholds. In the first variant of the model, *n*_s_ represented a fixed maximum number of samples the observer is able to use. In the second variant of the model, instead of estimating a fixed value for *n*_s_, we modeled the number of samples used as *n*_s_ = *n*_e_^*k*^, where 0 < *k* < 1, so that *n*_s_ always depends on the number of stimulus elements. These two models will be referred to as the simple model and the power model, respectively. Both models have three free parameters (σ_i_^2^ and σ_o_^2^ in both models plus *n*_s_ in the simple model and k in the power model). The model was fit for each observer individually by maximizing the log-likelihood of the parameter values given the data. One set of parameters was estimated for all conditions (a single model was fit to all noise conditions and stimulus sizes). For illustration in Figure 4, the power model was also fit to the averaged discrimination thresholds.

## Experiment 1: Effects of external noise and spatial properties

### Methods

**Figure 3.**
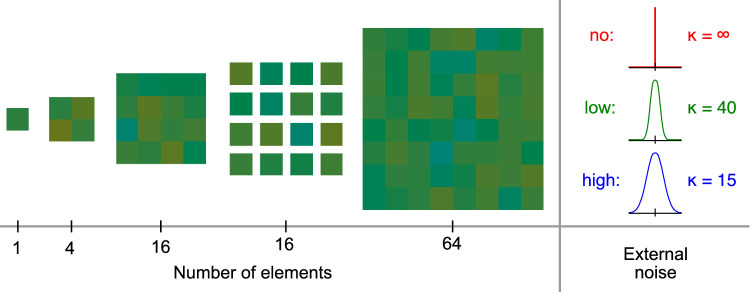
Experimental conditions for Experiment 1. The experiment included five different types of stimuli as seen on the left side of the figure and three levels of added noise for the element hues on the right. The one-element stimulus was only tested in the no-noise condition to avoid redundancy. Please note that the stimulus examples here and in subsequent figures are shown for illustration only and do not exactly match the stimuli in the experiment, which were color-calibrated and shown on a gray background.

Experiment 1 focused on estimating the number of stimulus elements observers use to judge the ensemble hue. We employed a factorial design in which the number of elements (1, 4, 16, 64) and amount of external noise in the hue of elements (no noise, low noise, high noise) served as independent variables (Figure 3). In a separate condition, we varied the distance between elements for the 16-element stimulus (abutting or separate elements). Also, the one element stimulus was only tested with the no-noise condition to avoid redundancy. This resulted in 13 experimental blocks, which were repeated twice in a random order for a total of 26 experimental blocks per observer.

Before the main experiment, the observers completed a practice run with a subset of nine experimental conditions, with a small number of repetitions. A short demo introducing the different stimuli and the task was shown before practice. A practice run was conducted at the beginning of each measurement session. Practice runs consisted of five repetitions for nine levels of the comparison stimulus for each of the nine experimental conditions included, resulting in 405 practice trials. In the main experiment, a single trial block contained 10 repetitions for the nine comparison levels for a given condition. Each block was run twice for the 13 conditions, resulting in a total of 2,340 trials for the main experiment. Experiment 1 took approximately two hours to finish, which observers completed in one or two sessions.

### Results and discussion

**Figure 4.**
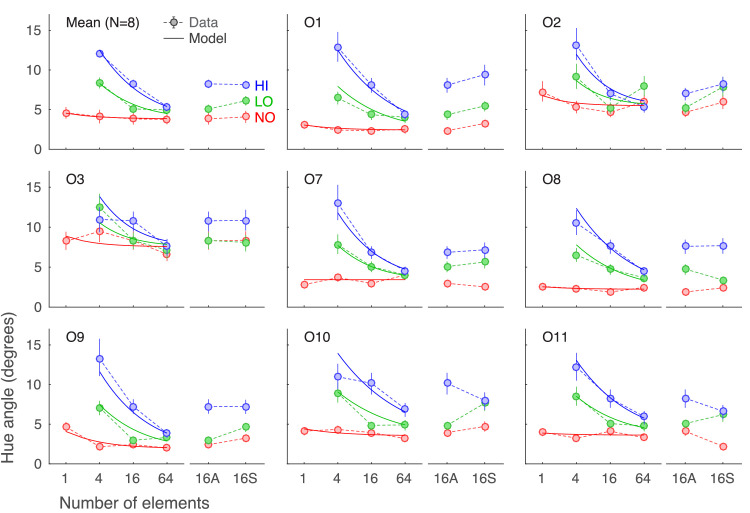
Discrimination thresholds for Experiment 1. The top left panel shows the average data over eight observers; the other panels show individual observer data. The x-axes indicate different set size conditions and the y-axes the discrimination thresholds in degrees of CIELAB hue angle. Different colors indicate different noise conditions: no noise (NO) in red, low noise (LO) in green, and high noise (HI) in blue. Symbols and dashed lines show the estimated discrimination thresholds from the data with error bars showing ±1 *SEM*. Solid lines show the model fits. The comparison between the 16-element condition with abutting (16A) and separated (16S) elements is shown on the right of each panel.

The main results for all eight observers who participated in Experiment 1 are shown in Figure 4 with measured discrimination thresholds indicated by the symbols and dashed lines and our model fit by solid lines. The results for the separated 16-element condition are shown next to the data from the abutting 16-element condition (16S and 16A) on the right of each panel. Figure 4 shows three main results: (1) The task was more difficult with more external noise, (2) performance improved as the number of elements increased, and (3) the improvement in performance with increasing number of elements was more pronounced at higher noise levels. To evaluate the robustness of these effects, we ran Bayesian repeated-measures ANOVAs with noise and set size as factors for set sizes 4, 16, and 64. There was strong evidence for the effect of noise (BF_10_ = 4 × 10^6^) and set size (BF_10_ = 630) when considered separately, but the strongest evidence was shown for the effect of both noise and set size together, along with their interaction (BF_10_ = 1.3 × 10^20^).

Our two variants of the noise models (the simple model and the power model) both fit the data well. The power model, however, did offer a slightly better fit for seven out of eight observers, evaluated by log-likelihood values. Thus, only the model fits of the power model are shown in Figure 4. With the power model, the exponent for the number of available elements to get the observer's effective sample size varied from 0.64 to 0.89 across observers. This means that the effective sampling ranged from a minimum of 2–3 in the 4-element condition to a maximum of 14–41 in the 64-element condition. The model also fit the averaged data well, giving an estimate of 0.83 for the exponent parameter, near the higher end of the estimates for individual observers.

It was recently suggested that hue averaging can be accounted for by a limited random subsampling mechanism using just two elements for averaging (Maule & Franklin, 2016). In contrast, not only did averaging performance increase from 16-element stimuli to 64-element stimuli in Experiment 1, but our modeling results pointed to a much higher estimate of maximum effective sampling size. Also, a slightly better fit was achieved when the number of samples observers utilized was allowed to vary with the number of elements available: The more elements in the stimulus, the more the observers can use. This is similar to what Dakin (2001) found with orientation and in line with a meta-analysis over several ensemble perception studies by Whitney and Leib (2018) suggesting a sampling rate of roughly the square root of displayed items.

To explore the effects of the spatial separation of elements, the abutting and separated versions of the 16-element condition were compared in all three external noise conditions. As illustrated on the right of each panel in Figure 4, performance was roughly the same whether the elements were abutting or not. A Bayesian ANOVA testing the effect of element separation was weakly in favor of no effect of separation (BF_10_ = 0.623).

Not finding a difference in performance between abutting and separated elements was somewhat surprising. First, based on previous research, we would expect that such contextual changes might affect both color appearance (Brown & MacLeod, 1997; Faul et al., 2008) and color discrimination (Krauskopf & Gegenfurtner, 1992). Kuriki (2004) also reported that observers perceived reddish colors from a mosaic with abutting squares of greenish colors. Second, the ensembles with abutting elements appear more like textures (or even objects), whereas the ensembles with separated elements appear more like the kinds of arrays commonly used in ensemble perception studies. That element separation does not matter suggests that “objecthood”—to the extent that our stimulus conditions manipulated it—may not be relevant for ensemble perception of hue.

## Experiment 2: Spatial properties

### Methods

**Figure 5.**
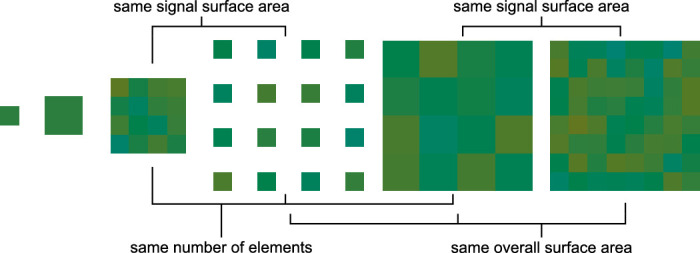
Experimental conditions for Experiment 2. The experiment included six different types of stimuli. The one element stimuli were only tested in the no-noise condition and acted mainly as a control. The other four stimuli were designed to share some of the aspects named in the figure while differing in others: number of elements, signal surface area, and overall surface area. They were presented only in the high-noise condition. Please note that these stimulus examples are shown only for illustration and do not exactly match the stimuli in the experiment. The experimental stimuli were carefully color-calibrated and shown on a gray background.

We found in Experiment 1 that performance improves with the number of elements. However, we kept the element size and spacing constant in Experiment 1, which led to stimulus size increasing with the number of elements. We designed Experiment 2 to directly test the role of the number of elements in relation to other spatial factors. Figure 5 shows the different conditions, which varied in terms of the number of elements, element size, and element spacing.

The different stimulus conditions were designed to delineate between the effects of the number of elements, total signal area, and total stimulus coverage of the visual field. The stimuli consisted of either 1-degree or 2-degree elements, so we measured the discrimination thresholds for these with no external noise to see if they are comparable (these are identical to the no-noise, one element, and four element conditions in Experiment 1). The four main stimulus conditions were (from left to right in Figure 5) a 16-element stimulus with abutting 1-degree elements, a 16-element stimulus with 1-degree elements separated by a 1 $\frac{1}{3}$-degree gap, a 16-element stimulus with abutting 2-degree elements, and a 64-element stimulus with abutting 1-degree elements. Of these, the first two and the last two had the same signal surface area, the first three had the same number of elements, and the last three had the same overall coverage of the visual field. In each of these main conditions, stimulus hues were drawn from the high-noise distribution of Experiment 1.

Similarly to Experiment 1, Experiment 2 consisted of a practice session and the main experiment, preceded by a short demo. The practice included all six experimental conditions (see Figure 5) with seven repetitions for each of the nine comparison levels, resulting in 378 trials. In the main experiment, the six experimental blocks were repeated twice in random order, resulting in 12 blocks. With each block having 10 repetitions for nine comparison levels, there were in total 1,080 trials per observer. The whole experiment took approximately one hour to finish, and all observers completed it in one session.

### Results and discussion

**Figure 6.**
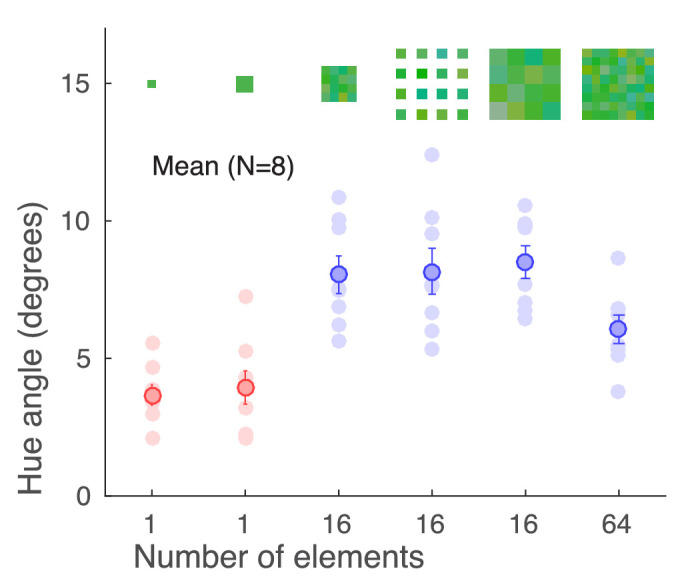
Discrimination thresholds in Experiment 2. Averages over eight observers are shown with thick closed circles; the unsaturated circles show the data for individual observers. The different experimental conditions are shown on the x-axis and indicated with insets above the data points, while the y-axis shows the discrimination thresholds (*SD*s, measured in hue angle). Error bars show ±1 *SEM*. Colors are as in Figure 4 (red = no noise, blue = high noise).

Figure 6 shows the results for Experiment 2. First, element size had little effect on performance, as shown by the similarity of the two leftmost data points; a Bayesian planned comparison *t* test was somewhat in favor of no difference (BF_10_ = 0.46). This is in line with the results from Experiment 1 where thresholds in the no-noise condition were little affected by stimulus size. The single elements thus seemed to provide equal information of hue. Next, we compared stimulus arrays with different element sizes. Despite different spatial manipulations, observers demonstrated equal average performance in all 16-element conditions. The only consistent effect on performance was due to the number of elements in the stimulus, with a significantly lower discrimination threshold average in the 64-element condition. A Bayesian one-way ANOVA with the three 16-element conditions and the 64-element condition as levels showed an overall difference (BF_10_ = 16.04). Planned comparisons showed moderate to strong evidence for the difference between the 64-element condition and all 16-element conditions (all BF_10_ > 9.90) and some evidence for no difference between the 16-element conditions (all BF_10_ = 0.36–0.38).

These results show, first, that the effects we saw in Experiment 1 were due to the number of elements and not differences in other spatial properties—namely, area—of the stimulus. Second, and more surprisingly, they suggest that the mechanism underlying ensemble perception for hue is insensitive to salient differences in the stimuli, such as the distance between individual elements and the size of the stimulus array, at least within the limits of the stimuli used here.

## Experiment 3: Effect of distribution shape

### Methods

In the third experiment, we asked whether changes in the shape of the noise distribution produce changes to observers’ spontaneous integration strategy. For the purpose of spontaneity, we avoided any mention of “mean” or “average” in observer instructions. Instead, observers were asked to answer according to what they perceived as “yellower” or “bluer” and were told that there was not necessarily a correct answer.

In this experiment, instead of performance, we were mainly interested in perceptual bias. Three different set size conditions (4, 16, and 64) were selected with two levels of noise (low and high). Each of these six experimental conditions was tested both with a skewed distribution and with a baseline condition with no skew. In the skewed noise conditions, element hues for the standard stimulus were drawn from a strongly skewed (skew ≈±.96, switched between observers) normal distribution with a standard deviation closely matching the von Mises κ values of 40 and 15 from other experiments (*SD*s were approximately 9.12 and 14.79 degrees of hue angle for low and high noise, respectively). The variances of the normal distribution were sufficiently small not to wrap around the hue circle. Hence, a normal distribution is practically identical to the von Mises distribution in this case. For observers who had already completed Experiment 1, baseline measures of response bias were taken from the corresponding measures (but with abutting elements). For other observers, a separate baseline measurement was conducted.

The comparison stimuli were similar to Experiments 1 and 2 except for having no added external noise; in other words, all the elements were of the same hue. The reason for this was that applying the same skewed distribution to both the standard and comparison stimulus would prevent detecting any perceptual bias, as any perceptual bias would apply equally to both stimuli. Also, using a normal distribution in the comparison stimuli might have prevented observers from learning the distribution characteristics of the standard stimulus. Because the comparison stimuli were of uniform hue, both the standard and comparison stimuli were presented with separated elements (separation of $\frac{1}{3}$ element size) to avoid having observers compare stimuli with very different edge information. In contrast, for baseline measurements, the comparison stimulus hues were drawn from the same distribution as the standard stimulus hues, similarly to Experiment 1.

The experiment consisted of a practice run and the main experiment preceded by a short demo. Practice runs had seven repetitions for nine comparison levels for all of the six experimental conditions, resulting in 378 trials total. For the main experiment, there were 12 blocks in total as the six experiment blocks were all presented twice. The observers first completed all six experimental blocks in a random order, after which they completed the same six blocks again, also in a random order. This was done to enable us to gauge possible learning effects. Each block had 10 repetitions for nine comparison levels, resulting in a total of 1,080 trials. The main experiment took observers approximately one hour. The additional baseline measurements shared all the aforementioned details and similarly took observers approximately one hour. Furthermore, because of a programming error, the experiment procedure only repeated the experimental blocks for the low-noise conditions four times each for the first four observers. After remedying the issue, three of the four observers returned for an additional measurement of the missing high-noise condition, which included 540 trials, but observer O3 only completed the low-noise condition.

### Results and discussion

**Figure 7.**
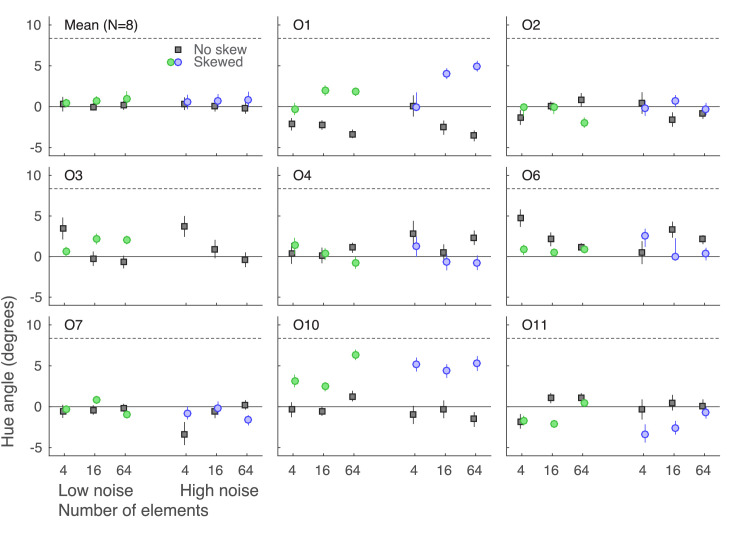
Perceptual biases in Experiment 3. The top left panel shows data averaged over eight observers, while the other panels show individual observer data. X-axes indicate different set size and noise conditions and y-axes indicate bias (point of subjective equality minus the standard stimulus mean, measured in degrees of hue angle). The solid line at the zero mark on the y-axis indicates the distribution mean and the dashed line the mode for the low-noise condition (high-noise mode is located at 13.54 on the y-axis, out of graph range). Gray squares indicate the baseline biases (with no skew) while green and blue dots indicate biases with skewed low and high noise, respectively. Error bars show ±1 *SEM*. Note that although different observers were assigned with either a positively or a negatively skewed noise distribution, the results are flipped to always have the distribution mode in the positive direction.

The results of Experiment 3 are visualized in Figure 7. The results were flipped for observers who had a positively skewed distribution in their measurements. This was done to always have the skewed distribution mode in the positive direction for the sake of comparison and statistical testing. Also note that only the mode for the low-noise distribution is visible in Figure 7, located at 8.36 on the y-axis. The mode for the high-noise condition is located at 13.54 on the y-axis and thus out of the graph range.

Only observer O10 showed consistent biases away from the mean in responses overall, and observer O1 only showed biases with larger set sizes. Other PSEs were located close to the distribution mean. A three-way repeated-measures Bayesian ANOVA with distribution skew, number of elements, and external noise as factors confirmed that responses were not significantly biased toward the mode (no model showed more evidence for the alternative hypothesis, and the models with all three factors and second/third-order interaction showed more evidence for the null, with BF_10_ below 0.07). The results show little difference from a simple averaging strategy where all elements are weighted equally.

Only two of our eight observers showed any sign of adjusting their strategy from simple averaging into discarding or downweighting outliers and/or upweighting most numerous hues, all of which would shift the observer's PSE from the distribution mean toward the mode. In these two cases, the most bias was seen with higher noise and larger set sizes, which is not surprising considering that these cases included more information about the shape of the distribution while also having a larger possibility for significant outliers. Arguably, as the measured biases were still far from the distribution modes, the results could be explained as these observers simply discarding only the most extreme outliers. Considering that six of the eight observers were effectively unbiased, discarding outliers does not seem to be a general strategy in this task.

## General discussion

Our results show that observers are effective in averaging hue information when the number of elements in a hue ensemble increases. We estimated the sample size used in averaging to be the number of displayed items to the power of 0.83, or roughly half of 64 elements. The advantages of averaging are especially visible with high external uncertainty about the element hues, but a simple noise model accounts for performance with all noise levels on a single-observer basis. Importantly, the improvement in performance is driven by the number of elements and not by stimulus or signal area. Finally, even with a strongly skewed hue distribution, most observers seem to spontaneously favor a simple averaging strategy.

Our main findings from Experiment 1 point toward much more effective sampling in hue integration than has previously been reported by Maule and Franklin (2016). This discrepancy, however, might be more superficial than it initially seems and is probably mostly explained by differences in the stimuli between the two studies. Instead of having genuine stochastic variation, the hues in the Maule and Franklin study were selected from a set of predefined hues separated by a number of steps in just noticeable hue differences. Further, their 16-element stimulus had only four of these distinct and discriminable hues, each repeated four times. Thus, if considering the number of *distinct* hues in the stimulus, the sampling rate estimated by Maule and Franklin is consistent with our modeling results, which would indicate roughly two samples for the four-element stimulus, as well as previous estimates from other feature dimensions when only a small number of items were available to the observer (Allik et al., 2013; Haberman & Whitney, 2009; Sweeny & Whitney, 2014). It is also possible that the small number of distinct hues in Maule and Franklin's study may have encouraged a more cognitive strategy: Tokita et al. (2016) proposed that observers employ different strategies when estimating summary statistics from small sets and similar strategies with larger sets.

We tested hue integration on a restricted range of hues, but considering that hue averaging is relatively accurate in several different hue categories (Kimura, 2018), we believe the present results generalize to other hue ranges, as long as the hue variation in the stimulus remains moderate. For large hue variations, rapid averaging of hue becomes inefficient (Maule & Franklin, 2015).

The estimated number of elements utilized by our observers clearly surpasses the commonly held limits of attentional (Scimeca & Franconeri, 2015) and working memory resources (Luck & Vogel, 2013), assuming each element was attended serially. Therefore, our results support a more global mechanism with distributed attention in averaging hue, which has been suggested in some form in ensemble perception of other stimulus types (e.g., Alvarez & Oliva, 2008; Chong & Treisman, 2003; Corbett & Oriet, 2011; Im & Halberda, 2013). We cannot directly rule out an alternative strategy, such as smart subsampling (i.e., directing sampling to the most meaningful targets), which would reduce the required number of samples and allow for focal attention (see Lau & Brady, 2018). However, smart subsampling would presumably affect the high-noise condition—where the hues are more variable—more than the conditions with low or no noise, whereas our noise model accounts for the data from all noise conditions with a single set of parameters. We thus consider smart subsampling an unlikely strategy for our observers.

Experiment 2 confirmed that the improvement in hue averaging was driven by the number of hue elements and not by stimulus area, signal area, or total stimulus border length. Furthermore, whether the elements abutted or not did not have a significant effect on the efficiency of hue averaging. This result held both with a small (Experiment 1) and a much larger gap size (Experiment 2). We find the lack of an effect surprising, because the gap manipulation dramatically altered the appearance of the stimulus: The stimulus with abutting elements appeared like a surface with a chromatic texture, whereas the stimulus with separate elements appeared like a typical ensemble stimulus array. One might assume that a unitary or objectlike stimulus is processed differently from an array, perhaps such that integration is more efficient (and discrimination performance better), but this was not the case. It is conceivable that the lack of effect of the gap manipulation was due to the simplicity of the stimuli: They consisted of simple flat shapes and thus were rather impoverished compared to, say, natural stimuli. Further, they did not contain variation in lightness or saturation, which may be more influential modulators for ensemble processing. The hue array was also the only item on the display. This may have led the observers to treat the stimulus with separated elements as a kind of texture, causing them to integrate hue over space regardless of spatial layout.

Experiments 1 and 2 showed that observers are efficient at integrating information over large hue ensembles for Gaussian distributions. In Experiment 3, we asked whether observers were sensitive to higher-order statistics of the hue ensemble by modifying the skewness, in addition to the standard deviation, of the hue distribution. It turned out that observers spontaneously opted for a simple averaging strategy, largely ignoring higher-level statistical information in the hue distribution. This may seem at odds with previous studies characterizing the integration of color information from non-Gaussian ensembles (Chetverikov et al., 2017; De Gardelle & Summerfield, 2011). Chetverikov et al. (2017) found that reaction times for finding a target in a search array differed as a function of how far the target color was from a previously learned distractor color distribution mean and that this pattern differed for Gaussian versus uniform distractor distributions. However, their task was intended to tap into *implicit* representations of color distributions and did not require that the observers form explicit estimates based on perceived hue. In contrast, we set out to examine whether observers would spontaneously form skewed representations in an explicit discrimination task (in other words, is the ensemble percept rich enough to support such inferences).

A more explicit higher-level representation of color distributions was implicated by De Gardelle and Summerfield (2011): They found that both the mean and variance of stimulus sets independently affected speed and accuracy in a color averaging task. Importantly, their observers downweighted or ignored items that were further from the decision criterion (i.e., the threshold between red and blue categories) and what they regarded as outliers (but see Van Den Berg & Ma, 2012).

This discrepancy between the present results with previous work may be due to an important difference in task structure. Our task had a comparison (decision criterion) varying from trial to trial and always presented after the standard stimulus. De Gardelle and Summerfield employed the category limit as a stable criterion over the whole experiment, making it more plausible for observers to cognitively focus on a certain, more limited color (or shape) area. This could have led observers to apply an explicit strategy of relying on the items nearest to the category boundary as a way to limit the cognitive demands of the task. Even in light of the results in Experiment 3, we do not argue that observers *could not* take the shape of the distribution into account if needed. We deliberately tested for a spontaneous strategy when making hue estimates and provided no feedback during the experiment. Had we measured performance and provided feedback, observers may well have learned to take the shape of the distribution into account. In the case of the two observers who showed a difference in mean estimates with skewed noise distributions, we cannot say whether they were discarding outliers, estimating distribution shape, or employing an altogether different strategy.

We chose to use simple two-dimensional stimuli in the interest of experimental control to characterize the principles of ensemble perception of hue. A logical next step is to extend these investigations to three-dimensional objects to address the important question of whether hue ensembles are processed similarly for objects as compared to non-objectlike stimuli or scenes. It is also important to note that, for the sake of simplicity, we varied our stimuli only in terms of hue while keeping saturation and lightness constant, but it is clear that natural stimuli contain variation in all three dimensions of color. A three-dimensional stimulus space introduces additional complexity into characterizing summary representations compared to many other stimulus types in the ensemble perception literature. For instance, in a task to match a uniform color to a 20 × 20 color mosaic (randomized from nine preset colors) by method of adjustment, Kuriki (2004) found that as the variation of colors in an ensemble increased, observers’ estimates drifted away from the colorimetric mean toward the most saturated color when all the elements were roughly within the same color category. Similar dependencies of ensemble estimates on color saturation were shown by Kimura (2018) and Sunaga and Yamashita (2007). Along with studying color ensemble percepts for natural shapes in future studies, it is important to investigate how ensemble encoding depends on all three color dimensions.

In conclusion, we found observers to be more effective at averaging hue distributions than previously thought. Surprisingly, spatial properties of the stimulus such as element size, array size, or element spacing did not affect averaging. Our results suggest that the hue of an object with varying surface reflectance is not determined from singular sample locations but averaged over larger areas, even when the stimulus is not spatially continuous. Furthermore, at least with simple two-dimensional arrays, observers seem not to be spontaneously sensitive to higher-order statistics when encoding ensembles with non-normal distributions. These results shed light on the discrimination and identification of multicolored stimuli and offer a firm basis to investigate spatial integration of color in more realistic scenes.
